# Oligodendrocytes interactions with glial cells and neurons in demyelinating disease: Oligodendrocyte interactions in demyelination

**DOI:** 10.3724/abbs.2025105

**Published:** 2025-08-13

**Authors:** Jiayi He, Qingqing Sun, Xiaowen Li, Ruoyan Du, Haoran Xue, Yuan Zhang, Xing Li

**Affiliations:** National Engineering Laboratory for Resource Development of Endangered Crude Drugs in Northwest China Key Laboratory of Medicinal Resources and Natural Pharmaceutical Chemistry (Shaanxi Normal University) The Ministry of Education College of Life Sciences Shaanxi Normal University Xi′an 710119 China

**Keywords:** oligodendrocytes, cell interactions, glial cells, demyelinating diseases, neuroinflammation

## Abstract

This review delves into the crosstalk network between oligodendrocytes and other glial cells in the context of demyelinating diseases. Oligodendrocytes, the myelin-forming cells in the central nervous system (CNS), are crucial for maintaining the function and integrity of axons and myelin sheaths. In demyelination pathologies, various factors hinder the normal differentiation of oligodendrocyte precursor cells, obstructing the myelin regeneration process, which is a primary barrier to therapeutic myelin repair. Emerging evidence highlights the critical role of glial cell interactions in CNS homeostasis and myelin regeneration, increasing interest in the treatment of demyelinating diseases. This article reviews the role of interactions between oligodendrocytes and other CNS glial cells in demyelinating and neurological diseases. Previous findings suggest that other CNS glial cells influence the survival and metabolic activity of oligodendrocytes through cell interactions, significantly affecting myelin formation and impacting demyelinating diseases characterized by myelin regeneration disorders. Targeted modulation of oligodendrocytes and their interactions with other cells at specific temporal stages may indicate a novel therapeutic direction for demyelinating diseases and offer fresh perspectives on the study of mechanisms and therapeutic approaches for related neurological conditions.

## Introduction

Glial cells, including oligodendrocytes (OLGs), microglia, and astrocytes, are the most numerous cells in the central nervous system (CNS). Each type of glial cells has a distinct function and plays various roles in the CNS [1]. They work in conjunction with neurons to maintain the homeostasis of the CNS through intercellular interactions [2]. Glial cells also exert direct or indirect effects on other cells in the CNS [ 3– 5]. Recent studies suggest that understanding the relationships among different cell types is essential for comprehending the pathogenesis of neurological diseases rather than focusing only on a single cell type [ 6– 8]. Elucidating the regulatory network among different types of glial cells is therefore crucial for obtaining a comprehensive understanding of the occurrence and progression of neurological disease.

OLGs are myelin-forming cells in the CNS [9]. They originate from oligodendrocyte precursor cells (OPCs) and undergo differentiation [10]. The primary function of OLGs is to produce myelin and facilitate the conduction of action potentials [ 11 – 13]. Additionally, OLGs secrete neurotrophic factors, promote the survival and function of neurons and other glial cells, and help maintain the dynamic balance of myelin in the CNS alongside other glial cells. OLGs play a crucial role in the regeneration of myelin following injury [14]. In animal models of demyelinating disease, OLGs can regenerate myelin, but this regeneration process may be temporary or fail under pathological conditions [15]. Therefore, it is essential to systematically understand and study the dynamic process of OLGs during myelin injury and the body’s subsequent response.

It is increasingly recognized that interactions between different types of glial cells play important roles in regulating the health of myelin, which is the protective covering that surrounds nerve fibers [16]. OLGs, the cells that produce myelin, are potential targets for disease treatment because they play a critical role in maintaining the health of myelin. Hence, the impact of other glial cells on myelination is predominantly achieved by regulating the activity of OLGs. For instance, astrocytes help regulate the amount of cholesterol in the brain, which can affect the survival of OLGs and support the regeneration of myelin. Astrocytes also provide different types of assistance for myelin health during active injury than they do during regeneration after damage has already occurred [17]. Microglia, another type of glial cell, are crucial for maintaining myelin health. They help refine the myelin sheath through phagocytosis during growth and development, which is essential for the normal development, proliferation, and maturation of OLGs. Under disease conditions, microglia are concentrated in lesion areas and can produce proteins that affect the development of OPCs [18]. As OLGs are highly susceptible to microglia-derived factors, the influence of microglia gathered in the lesion area on OLGs cannot be ignored. High levels of proliferation and activation of microglia occur mainly in the early stage of multiple sclerosis (MS), especially in the active part of demyelination. This is more likely to occur during the early stages before the onset of pathology rather than during the later recovery stage. These findings suggest that research into microglia-OLGs should focus more on the early stages before the onset of pathology rather than the later recovery stage [19]. Effective communication between OLGs and neurons is essential for the maturation of neural circuits, although it is not yet fully understood. The rapid transmission of action potentials (AP) depends on the insulating properties of myelin [20]. The origin of neurons with myelinated axons and the specificity of OLGs in the myelination of particular neurons and axons are key factors in this process. For myelin to be produced and properly localized, efficient communication between neurons and OLGs is essential. In the neocortex, a single oligodendrocyte can myelinate multiple axons [21]. These findings highlight the critical role of OLGs in the interactions with other cell types during demyelinating diseases.

The myelin sheath is an essential part of the nervous system and consists of a tubular outer membrane composed of myelin that surrounds axons [22]. In the CNS, OLGs produce the myelin sheath, whereas in the peripheral nervous system, Schwann cells are responsible for its generation [ 23, 24]. Myelin formation is a complex process that depends on glial interactions and is necessary for the smooth transmission of information in the CNS [ 25, 26]. It also provides metabolic and nutritional support for neurons and can guide axon regeneration when axons are damaged. Demyelinating disease is a pathological phenomenon caused by damage to the myelin sheath, which cannot be replenished promptly, leading to a decrease in or even complete loss of myelin content in the nervous system [27]. This leads to dysfunction in the body that hinders normal physiological activities. The elimination of genes in OLGs leads to axonal damage, indicating that support from OLGs is essential for axons [28]. Under normal physiological conditions, OPCs can differentiate into OLGs and then produce a myelin sheath to maintain myelin plasticity and stability. However, under pathological conditions, the signal for myelin loss fails to trigger myelin formation in differentiated OLGs at the injured site. Myelin regeneration requires the reactivation of OPCs differentiation via the myelin formation pathway [ 29, 30]. Unfortunately, activated OPCs often accumulate at the edges of the demyelinating area and struggle to reach the injured interior [31]. Additionally, the metabolism of OPCs in the demyelinating CNS is compromised [32], making myelin generation challenging. In the demyelinating injury microenvironment, the aggregation of inflammatory cells leads to the production of pro-inflammatory cytokines. The reduced expression of neurotrophic factors, along with the accumulation of myelin regeneration inhibitors such as Lingo-1, NogoA, etc. [ 33, 34], further inhibits the differentiation of OPCs into mature OLGs [35]. As the condition progresses, the myelin sheath of the damaged part cannot be replenished in time, resulting in the development of demyelinating diseases.

Building on the understanding of demyelinating diseases and their impact on the nervous system, as outlined in the previous section, the goal of this paper is to delve deeper into the recent literature to summarize the intricate mechanisms of communication between OLGs and other cellular components within the CNS. Specifically, we aimed to investigate how these interactions are modulated over time and space and to explore their significance in both the progression of demyelinating diseases and the potential for therapeutic intervention.

## Demyelinating Diseases

Demyelinating diseases are complex nervous system diseases that occur when the body fails to replenish the myelin sheath in time after it is damaged. This leads to a reduction in or total absence of myelin in the nervous system, which affects normal physiological activities. The onset of these diseases is influenced by various factors and signaling pathways, which lead to various pathological phenomena. In demyelinating diseases, OLGs play a crucial role in demyelinating disease, with and their dysfunction is the main cause of these conditions [ 36, 37]. There are several common demyelinating diseases, including MS, neuromyelitis optica (NMO), and Guillain–Barré syndrome (GBS). Although these diseases have different clinical manifestations and pathological features, they all share the common features of OLGs damage and myelin destruction. For example, MS is characterized by macrophage-enriched demyelinating lesions in the white matter, with relatively preserved axonal integrity and the occurrence of reactive glial scarring [ 38, 39]. Unlike other presentations of demyelinating diseases, MS is clinically characterized by early relapse with neurological and radiological deterioration and a fluctuating course of remitting episodes. Currently, there is more evidence supporting the primary degenerative cause [40]. Myelin repair or regeneration is a common and relatively effective process in the early stage of MS, and the re-emergence of OLGs within damaged lesions associated with early myelin regeneration is expected to occur. However, in patients with advanced MS, myelin regeneration is rare or absent [ 41, 42]. Unlike the demyelinating diseases described above, for hereditary demyelinating diseases such as Pelizaeus-Merzbacher disease (PMD), genetic mutations in the *PLP1* gene can vary widely, including duplications, missense, and deletion mutations [ 43 , 44]. It is characterized mainly by a loss of myelination, leading to neurodevelopmental delays and generalized hypotonia. In cases of severe (congenital) PMD, patients may experience dyspnea and nystagmus at birth, followed by neurodegeneration and premature death in adolescence. This genetic trait makes it difficult to predict disease severity and progression on the basis solely of genetic testing. Thus, under current conditions, some research has focused on promoting remyelination through various strategies, such as stem cell therapy and gene therapy, but these methods are still experimental [ 45, 46]. Gene therapy for PMD faces barriers, such as how to efficiently deliver the corrected gene to affected cells and ensure long-term expression of functional proteins, which is a problem that needs to be addressed. The demyelinating diseases that have been mentioned are closely connected with oligodendrocyte lineage cells. To study these diseases, comprehensive knowledge of the mechanisms of OLGs differentiation and remyelination is necessary. Various complex factors have the potential to disrupt the normal function of OLGs in the lesioned microenvironment. Cell-to-cell communication, which is essential for coordinating responses in this environment, plays a crucial role in the process of OLGs maintenance and repair.

**Figure 1:**
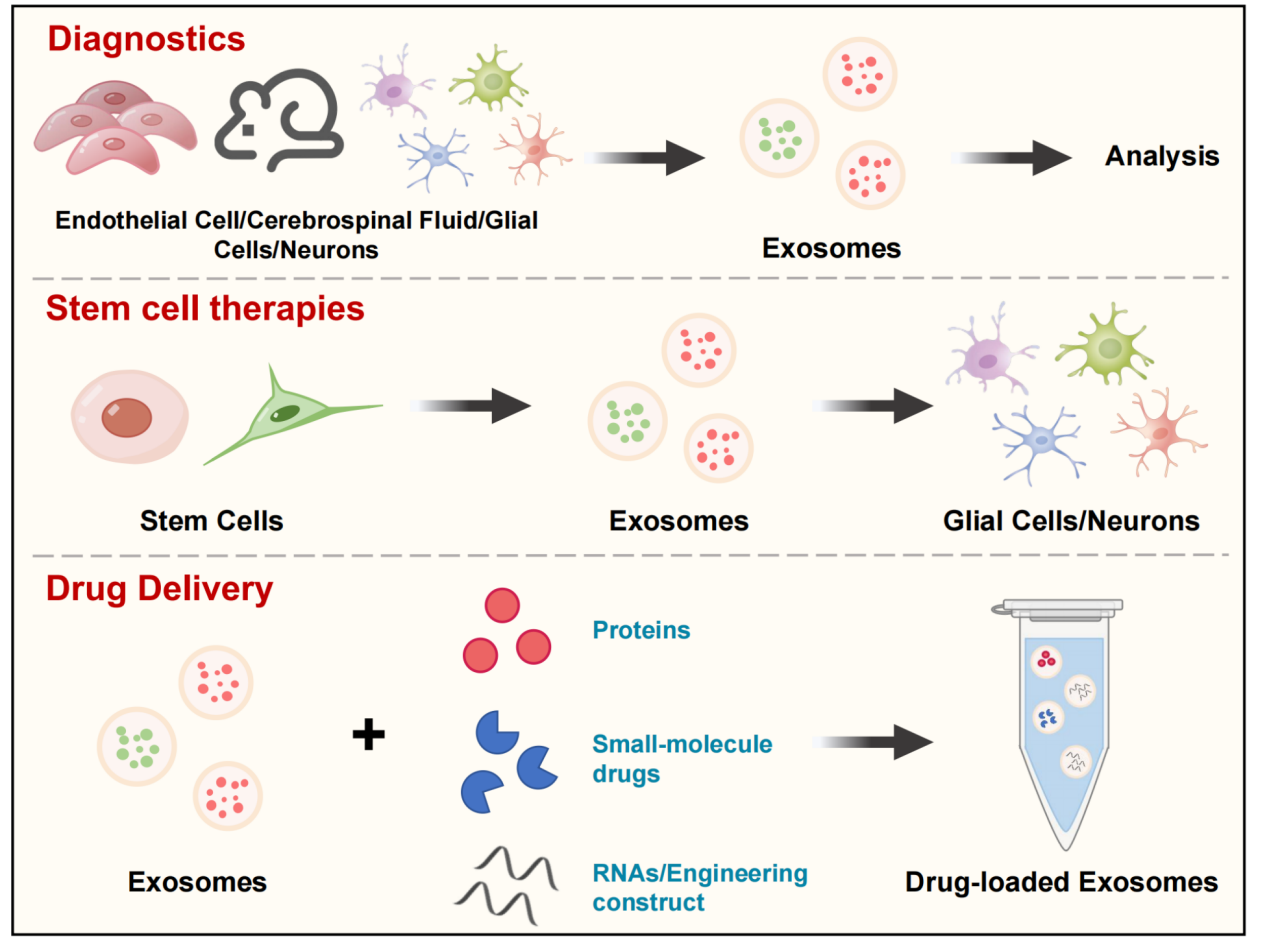
Figure 1 The application of exosomes in demyelinating diseases Exosomes play a significant role in demyelinating diseases, and the levels of exosomes released from glial cells, neurons, endothelial cells, and CSF can serve as therapeutic or diagnostic approaches for these conditions. Stem cell-based therapies offer a promising avenue, where exosomes derived from stem cells can facilitate repair following myelin loss. Engineered exosomes, capable of carrying small-molecule drugs, proteins, and nucleic acids, can achieve targeted therapy at the cellular level, highlighting their therapeutic advantages as delivery vehicles.

Currently, there is no universally effective treatment for all types of demyelinating diseases. Although drugs such as fingolimod and dimethyl fumarate are used for the clinical treatment of MS, these drugs primarily modulate the immune response, which can help manage symptoms and slow disease progression in some cases of MS [ 47– 49]. However, their impact on OLGs differentiation, which is critical for the repair phase of advanced demyelinating diseases, is not well understood, particularly as inflammation subsides [6]. In addition to these approaches, stem cell therapy, gene therapy, and exosome-based treatments have also demonstrated promising therapeutic effects. Adeno-associated virus vectors have emerged as valuable tools for gene therapy in neurological disorders because of their extensive biodistribution with *in vivo* delivery, lack of integration into the host genome, and stable expression [50]. Various delivery methods, including intravenous, intramuscular, intrathecal, or intra-neural injection and even injection into the dorsal root ganglia, have gradually shown great therapeutic potential for plasmid and viral vector delivery [51]. Stem cell therapy has been recognized as a cutting-edge field with significant potential for treating MS [52]. Characterized by their inherent self-renewal and pluripotency, stem cells can be transplanted to differentiate into various neural cell types or secrete neurotrophic factors [ 53, 54]. This approach holds promise for regenerating damaged neural tissue, modulating immune responses, and creating an environment conducive to endogenous repair mechanisms. Additionally, exosomes derived from different sources have shown robust therapeutic capabilities in treating demyelinating diseases ( Figure 1) [ 55, 56]. Exosomes not only serve as carriers for intercellular communication but also transport disease-specific biomarkers in patients with MS, thereby validating their potential as diagnostic biomarkers [ 57 , 58]. Moreover, exosomes derived from stem cells have been demonstrated to promote remyelination. Given that exosomes naturally participate in the exchange of biomolecules between cells, they hold great potential as novel drug delivery vehicles, especially for the delivery of biotherapeutic agents [59]. Today, various engineered exosomes loaded with proteins, small-molecule drugs, and RNAs have undergone extensive development [ 60– 62].

Some evidence suggests that other glial cells in the vicinity may influence the differentiation of OLGs [63]. In-depth studies of the molecular mechanisms and cellular interactions involving OLGs could reveal novel targets for the development of more effective therapeutic strategies against demyelinating diseases.

## The Manner of Cross-Talk

As the basic unit of life, cells perform various essential functions that rely on interactions between them. Interactions between cells can be categorized into four main modes: (1) Chemical signal secretion: Cells communicate by secreting chemical signals such as small molecules, peptides, proteins, glycoproteins or lipids. This is a common way for multicellular organisms to communicate. Chemical signals transmit information between cells and regulate their physiological activities [18]. For example, in synapses, neurons release neurotransmitters through the presynaptic membrane, which bind to receptors on the postsynaptic membrane and thus transmit information. (2) Direct contact: Adjacent cells communicate with each other through direct contact. This communication mode mainly transmits information through the interaction of receptors present on the cell surface with ligands present in neighboring cells. For example, OPCs contact their processes on neuronal somata in a neuronal activity-dependent manner. OPC-neuron contact facilitates the exocytosis of neuronal lysosomes, thereby influencing neuronal metabolism and senescence [64]. (3) Gap junctions: These junctions form between adjacent cells and allow the exchange of small molecules and ions, facilitating the coordination of cellular physiological activities [ 6, 65]. These channels are formed by channel-forming proteins that are densely packed in the spatial microdomains of the plasma membrane [66]. When open, gap junction channels provide conduits for the direct exchange of small molecules and metabolites, thereby enabling communication between neighboring cells. Rapid electrical current transmission is mediated by gap junction channels between adjacent cells. These channels provide a low-resistance pathway for the propagation of presynaptic currents to the postsynaptic regions of electrical synapses in neurons [67]. Additionally, gap junctions facilitate the diffusion of signaling molecules, allowing calcium ions and their subsequent cytotoxic death factors to affect neighboring cells via these channels [ 68 , 69]. Furthermore, intercellular calcium wave propagation can be triggered by focal mechanical, electrical, or hormonal stimuli and is coordinated by the diffusion of IP3 through gap junctions between cells to orchestrate overall cellular responses. Accumulating evidence suggests that some connexin (Cx) channels are permeable to certain soluble second messengers, amino acids, nucleotides, and glucose, as well as their metabolites. (4) Vesicle delivery: Recent research, including our own, has revealed that vesicle trafficking can regulate cell‒cell communication, representing a new method of cross-talk [70]. Exosomes, which are bilayer lipid membrane structures secreted by cells, serve as primary vehicles for information transmission through vesicle transport. These vesicles contain RNA and protein and participate in various physiological activities. All types of cells in the central nervous system release exosomes, which play a role in regulating intercellular communication [71]. Although the formation and stability of neurites are affected by complex mechanisms, exosomal communication constitutes crucial mechanisms through which these structures are governed [72]. OLGs-derived exosomes, in particular, can be internalized by neurons in response to neuronal signals, exhibiting neuroprotective properties and potentially affecting other cell activities in the microenvironment [73]. Given their biocompatibility, low immunogenicity, and ability to cross the blood-brain barrier, exosomes show great potential as carriers for drugs, including protein and nucleotide drugs [ 74– 76]. We are also exploring their role in myelin regeneration and as potential treatments for demyelinating diseases.

**Figure 2:**
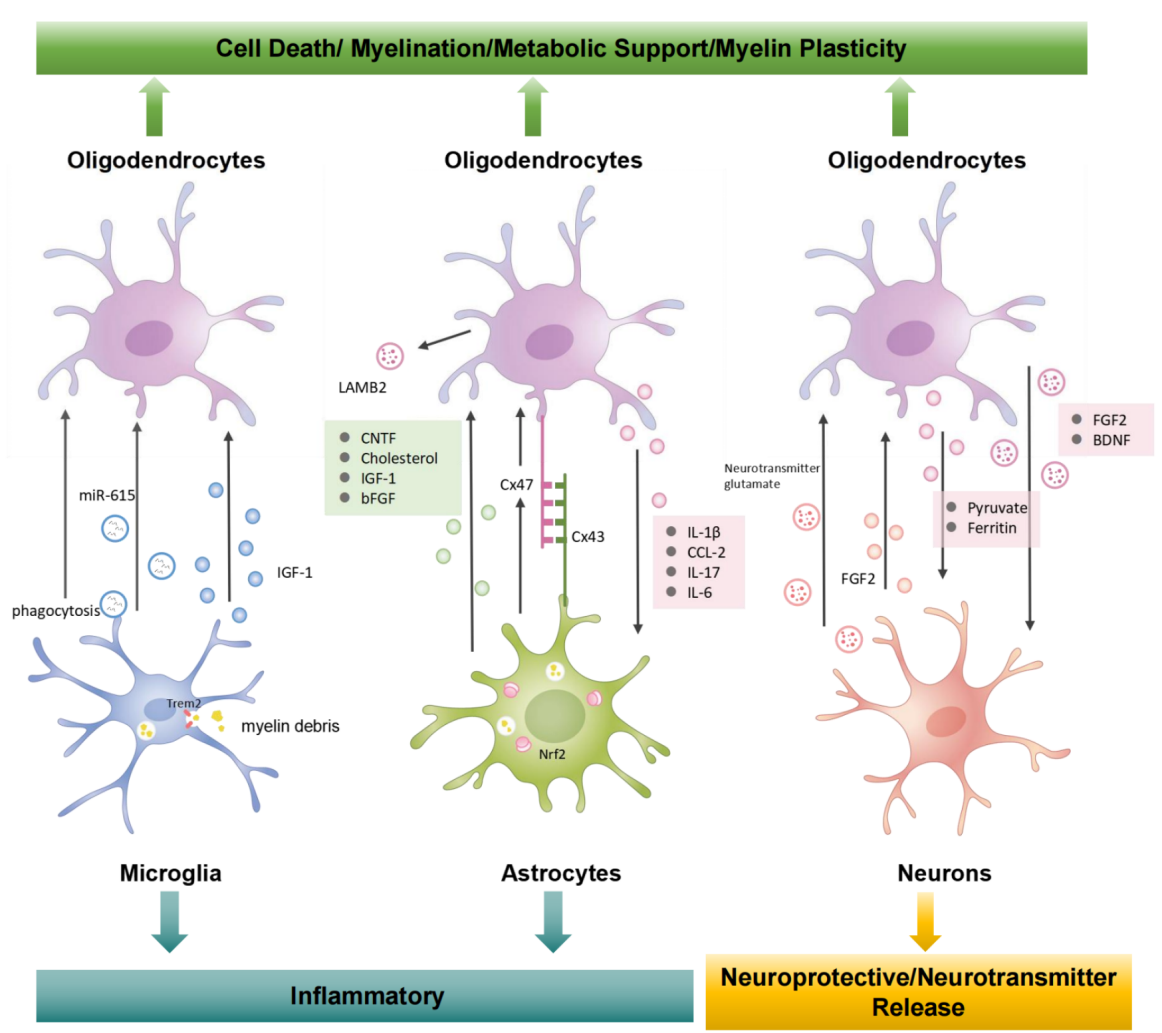
Figure 2 Schematic model of the mechanisms in the oligodendrocytes-glial cells crosstalk Crosstalk is essential for oligodendrocytes survival, differentiate and mature. Highly complex crosstalk networks control oligodendrocytes development and CNS myelination. Bidirectional communication between oligodendrocytes and other glial cells, as well as neurons, regulates their responses during CNS inflammation.

The coordination of processes involving multiple types of glial cells in the brain depends on communication between glial cells and the signals that convey information between cells ( Figure 2). Glial cells regulate the activity of nearby neurons by releasing signaling molecules and other substances, functioning similarly to neural circuits [77]. Cells in the human body can also communicate through direct interactions between their cell membranes, allowing for complex and well-controlled signaling processes. In the CNS, this membrane-to-membrane communication is key to establishing local and functionally distinct signal transduction pathways that regulate various biological processes.

The brain is a highly intricate biological system that is influenced by genetics, the environment, and various other factors. It comprises numerous neurons and glial cells that communicate through electrical and chemical signals, allowing for rapid and precise transmission of information. These interactions occur on the millisecond scale and adjust dynamically to changes in the environment and behavior. They manifest across different temporal and spatial scales, making it challenging to fully understand their effects on brain health and disease. Extensive and in-depth research is necessary to unravel this complex system.

An increasing number of studies support the notion that modulating the interactions between glial cells and neurons represents a crucial therapeutic strategy in the treatment of demyelinating diseases ( Table 1). For example, the interaction between microglia and astrocytes has significant pathophysiological implications for the evolution of NMO lesions. Chen *et al* . [91] developed an informative murine model of NMO by infusing AQP4-specific IgG (AQP4-IgG) continuously into the spinal subarachnoid space without exogenous complement. They reported that microglia are activated by signals emanating from AQP4-IgG-activated astrocytes. Markoullis *at al*. [92] reported the loss of O/A channels in the context of chronic inflammation and astrogliosis, favoring increased A/A connectivity, which may represent a relevant mechanism contributing to MS progression. These studies demonstrated that critical crosstalk is a potential target for therapeutic intervention.

**Table:** **Table 1** Drugs that act on the interaction between glial cells and neurons

Drug Names	Target	Profile	Reference
Luteolin	A-O	Inhibition of the Nrf2 pathway in astrocytes via luteolin promotes cholesterol efflux from astrocytes, thereby influencing the survival of OLGs and remyelination.	[17]
FSD-C10 (a Fasudil derivative)	M-O	The conditioned media from FSD-C10 stimulates microglia promoted OPCs survival and OLGs maturation.	[78]
Azithromycin	M-O	Azithromycin protected OPCs against LPS-microglia conditioned medium-induced damage.	[79]
FTY720	O-N	The downregulation of the growth factor BDNF in α-synuclein (αSYN)-treated OLN-93 OLGs was counteracted by FTY720, possibly pointing toward a critical role for this sphingosine analog in oligodendrocyte–neuron communication.	[80]
A-N	FTY720-phosphate (FTY-P) induced neuroprotective factors (leukemia inhibitory factor (LIF), interleukin 11 (IL11), and heparin-binding EGF-like growth factor (HBEGF)) in human astrocytes. FTY720 modulates the microenvironment in the brain via effects on astrocytes.	[81]
Dimethyl Fumarate (DMF), Monomethylfumarate (MMF)	M-O	DMF and MMF treated microglia supernatants led to an enhanced proliferation of OPCs.	[49]
DMF	M-N	DMF reduces microglia-mediated neurotoxicity. Differentiated HT22 hippocampal neurons were exposed to conditioned medium (CM) derived from microglia stimulated with LPS, in the absence of DMF. After 24 hours of exposure, a significant reduction in cell viability was observed in neurons incubated with CM from LPS-activated microglia.	[82]
Glatiramer Acetate (GA)	M-OPCs	GA has direct effects on the CNS, modulating microglia and producing microenvironmental changes with long-lasting effects on OPCs proliferation and maturation.	[83]
IFN-β	M-N	IFN-β is neuroprotective against the toxicity induced by activated microglia in cortical neurons and microglia cocultures.	[84]
Aripiprazole, Minocycline	M-O	Aripiprazole and minocycline may reduce OLGs damage caused by microglia activation.	[85]
Minocycline	M-OPCs	Minocycline-mediated inhibition of microglia activation impairs OPCs responses and remyelination.	[86]
Sulfasalazine (SF)	M-O	SF inhibited production of M1-like factors TNF-α and INF-γ in microglia, and thereby promoted the differentiation of OLN-93 OLGs.	[87]
Aspirin	A-O	Aspirin-treated astroglial CNTF is capable of increasing myelin-associated proteins in OLGs.	[88]
Curcumin	M-O	Curcumin protects preoligodendrocytes from activated microglia, has a protective effect on infection-driven white matter injury.	[89]
Fluoxetine	M-O	Fluoxetine inhibits OLGs cell death by inhibiting microglia activation and p38-MAPK activation, followed by pro-NGF production after spinal cord injury.	[90]

## OLGs Crosstalk with Other Glial Cells and Neurons

### Interaction between OLGs and astrocytes

Astrocytes are a type of glial cell that has a star-like shape. Derived from neural embryonic progenitor cells, astrocytes originate from the cavity of the embryonic nerve tube. Astrocytes are the most abundant type of glial cell and play an important role in synaptic formation, neurotransmitter transmission, cholesterol metabolism, fatty acid β-oxidation and CNS homeostasis [ 93, 94]. They have also been identified as key players in neurodegenerative diseases [95]. Astrocytes can promote the repeated firing of neurons by buffering excess K ^+^ and glutamate outside the cell to maintain the pH at a stable level. Additionally, the water channel aquaporin-4 (AQP4) in astrocytes regulates the flow of soluble molecules from cerebrospinal fluid into brain tissue and modulates cerebral hemodynamics [ 96, 97]. Astrocytes are of various types and are often divided into two categories: type I and type II. Protoplasmic astrocytes, also known as type I astrocytes, are located in gray matter, surround synapses and modulate synaptic transmission. They also surround blood vessels to promote synaptic and blood-brain barrier functions, respectively. On the other hand, fibrous astrocytes or type II astrocytes are found in the white matter. They contact the Ranvier lymph nodes and blood vessels [3]. During the pathology of MS/experimental autoimmune encephalomyelitis (EAE), reactive astrocytes extend into adjacent tissue to participate in lesion expansion. However, they also work to combat inflammation, limit tissue damage, and support the repair process [98]. These theories suggest that astrocytes could be viable targets for treating demyelinating diseases.

#### Astrocyte–OLGs interactions provide metabolic support to supply necessary nutrients to promote the survival and function of OLGs

The interaction between astrocytes and OLGs is crucial for the formation of the myelin sheath. Astrocytes are believed to play a vital role in supporting the survival and function of OLGs, and lipid exchange from astrocytes to OLGs provides critical metabolic support for OLGs, which in turn affects myelin. The metabolic exchange between these two cell types is thought to be crucial in the development of leukodystrophy associated with astrocyte dysfunction [99]. In focal lesions resulted from myelin damage in the mouse corpus callosum induced by the myelin toxin lysolecithin (LPC), the activation of Nrf2 in astrocytes induces OLGs death and prevents remyelination. Cholesterol availability is a rate-limiting factor in myelin production. Exogenous cholesterol increases oligodendrocyte differentiation [100]. Astrocytes can also regulate OLGs survival and support developmental myelination through the efficient transport of cholesterol, but this process is impaired by Nrf2 activation [ 17, 101, 102]. In an animal model of MS, the cholesterol synthesis pathway is downregulated in astrocytes of the cerebellum and spinal cord [103], which may lead to impaired differentiation of OLGs following demyelination.

#### Role of astrocyte–OLGs interactions in regulating the differentiation of OLGs

A heterotypic gap junction consisting of connexin 47 (Cx47)-Cx43 or Cx32-Cx30 can be established between astrocytes and OLGs [104], which influences myelination by transporting contents or affecting the K ^+^ concentration. In MS gray matter, the distinct elevation of astrocytic connexins may reflect active communication between astrocytes and OLGs [92]. This connexin located on the surface of astrocytes induces the secretion of exosomes that carry LAMB2 by OPCs via Cx47 to upregulate cyclin D1, thereby accelerating OPCs proliferation [105].

Astrocytes also secrete neurotrophic factors such as insulin-like growth factor 1 (IGF-1) and ciliary neurotrophic factor (CNTF), which also prevent OLGs apoptosis through the PI3K/AKT signaling [106]. Basic fibroblast growth factor (bFGF) is a growth factor produced by astrocytes that works synergistically with the extracellular matrix deposited by astrocytes to enhance OLGs elongation [107]. Astrocytes can selectively influence OLGs. These cells release PDGF and additional LIF-like cytokines to promote the OLGs survival. They can also keep OLGs in a mature myelinogenic state, promoting the formation of myelin [108]. Astrocytes play an indispensable role in maintaining the balance between myelin regeneration by Schwann cells and OLGs. This balance of regeneration is influenced by astrocytes through signal transducer and activator of transcription 3 (Stat3) signaling. In a mouse model of conditional knockout of phosphorylated Stat3 in astrocytes, OLGs-mediated myelin regeneration decreased, whereas Schwann cell myelin regeneration increased in damaged knockout mice compared with control mice. This results in increased myelin regeneration in Schwann cells and decreased myelin regeneration in OLGs [109]. Iron uptake and release from astrocytes are associated with OPCs and OLGs iron homeostasis. Iron efflux from astrocytes, which is mediated by iron-exporting proteins, has been shown to be associated with OPCs maturation and myelin regeneration [110].

Astrocytes release exosomes that enhance the chemotaxis of OPCs, improving their differentiation and migration, particularly under conditions such as ischemia, while also inhibiting their proliferation under severe hypoxia [111]. However, exosomes from aged astrocytes have a negative effect on OLGs maturation. In addition, astrocytes can stimulate OPCs proliferation by increasing the production of exosomes in OPCs through ITGB4-mediated cell adhesion [112]. Thus, it can be inferred that exosomes play an important role in intercellular communication between OPCs and astrocytes [106].

#### Role of astrocyte–OLGs interactions in immune regulation

However, in addition to inhibiting apoptosis, several studies have shown that activated astrocytes secrete various substances, such as TNFα, Fas ligand (FasL), and glutamate, which leads to the apoptosis of OLGs, reduced myelin regeneration, myelin clearance, and subsequent neuronal death. This process is thought to amplify and even initiate CNS autoimmunity responses. On the other hand, astrocytes also promote the neuroprotective function of OLGs by recruiting OPCs to the site of inflammation through the secretion of CXCL1, IL-8, and CCL-2. Additionally, these astrocytes produce CNTFs, which promote the differentiation of OPCs into mature myelinating cells. As a result, this activity increases myelin regeneration in inflammatory regions of the CNS, ultimately helping to restore nerve conduction.

OLGs have traditionally been considered immunologically inert and merely serve as bystanders in the pathological responses of neuroglia and immune cells. However, recent studies have shown that OLGs play an active role in immune regulation of the central nervous system. They participate in phagocytosis, antigen presentation, and the activation of memory and effector CD4 ^+^ T cells. Furthermore, OLGs secrete pro-inflammatory cytokines such as IL-1β, CCL-2, IL-17, and IL-6, which trigger NF-κB signaling and promote pro-inflammatory response in astrocytes [113].

In summary, astrocytes and OPCs engage in a complex interplay within the CNS, where astrocytes have both detrimental and beneficial effects on OPCs and myelin health. The balance of these interactions, modulated by various factors, including cytokines, growth factors, and exosomes, is critical for maintaining CNS homeostasis and could be pivotal in the development of therapeutic strategies for demyelinating diseases and other neurological disorders.

### Interaction between OLGs and microglia

Microglia are resident immune cells in the CNS that originate from the yolk sac. Microglia promote the pruning of developing neurons that are in excess or flawed during synapse formation [114]. Microglia in the CNS are highly dynamic [18] and can switch phenotypes in response to injury, stress and neurodegeneration [115]. Considering microglial diversity, some papers categorize microglia into M1 and M2 polarization. The M1 polarization is challenged by interferon-γ (IFN-γ) and lipopolysaccharide (LPS) and produce tumor necrosis factor-α (TNF-α), interleukin-1β (IL-1β), IL-6, IL-12, nitric oxide (NO), and reactive oxygen species (ROS), which are neurotoxic substances that promote inflammation and aggravate nerve damage. The M2 polarization also secretes cytokines such as IL-4, IL-10, transforming growth factor-β (TGF-β), vascular endothelial growth factor (VEGF), and brain-derived neurotrophic factor (BDNF). These cytokines can exert an anti-inflammatory response, phagocytose damaged neuronal fragments, and promote nerve repair [ 116, 117 ]. The proportion of each phenotype may vary depending on the stage of a neurodegenerative disease [ 118, 119 ]. In addition, microglia can release nutrient factors into the surrounding environment to support the formation of neuronal circuits and promote neuronal survival. Microglia are highly specialized cells within the CNS that exhibit functional autonomy and heterogeneity. This diversity allows them to monitor and communicate with multiple compartments of different neurons and various cell types [120]. The interactions between microglia and neurons, as well as the surrounding chemical environment, can influence signal transduction and the nature of these interactions [121]. Glial cells such as astrocytes and OLGs form gap junction channels, establishing stable intercellular relationships [ 22, 122 ]. These channels facilitate the exchange of ions and small molecules, contributing to the coordination of cellular activities.

#### Role of microglia‒OLGs interactions in regulating the differentiation of OLGs

Microglia play a crucial role in the development of OLGs by producing IGF-1. IGF-1 promotes the differentiation of pluripotent neural progenitors into OLGs [123] and provides long-term protection for immature OLGs from glutamate-mediated apoptosis. Activin-a is another important mediator that promotes OLGs differentiation by microglia [3]. Microglia also promote OPCs differentiation through hemagglutinin-3 and cholesterol precursors while regulating the balance of OLGs. Activated microglia release various substances that cause inflammation, which are mainly produced to fight off invading pathogens; however, they also damage nearby glial cells and neurons. In an inflammatory environment, OLGs are particularly vulnerable to the effects of these substances because of their high metabolic activity and energy requirements. This can lead to the production of low-quality myelin, which is worse than having no myelin at all [124]. Consequently, various neurological diseases can develop. *In vitro*, when exposed to LPS, microglia challenged with LPS can inhibit myelin regeneration by preventing OPCs proliferation and inducing OPCs death [125]. Although macrophages and microglia cause damage to OLGs and myelin during inflammation, they are also instrumental in wound healing, regeneration, and repair in the CNS. According to some studies, microglia may play a critical role in recruiting OPCs to demyelinated regions, as well as in promoting the proliferation and maturation of OPCs, leading to the restoration of myelin. Research on animal models has demonstrated that removing or blocking the activity of microglia has a negative effect on the repair process, which highlights the importance of the interaction between microglia and OLGs in myelin regeneration [126].

The inflammatory exosomes secreted by microglia also inhibit OPCs differentiation into OLGs and myelin regeneration. Microglia-derived exosomes containing endocannabinoids are conducive to OPCs migration and differentiation and promote myelin repair [127]. In addition, exosome-carried miRNAs also inhibit intrinsic OPCs differentiation-related transcription factors. We recently identified miR-615, a crucial component of exosomes released from activated microglia, as a candidate miRNA that silences MYRF, thus blocking myelination/remyelination. Furthermore, the administration of an AAV-pIba1-miR-615 sponge to EAE mice alleviates the disease development and promotes remyelination [70].

#### Microglia regulate OLGs through the process of myelin clearance

The myelin sheath is a flexible structure that continuously changes in response to various factors in the surrounding environment. OLGs generate a significant number of new myelin sheaths along axons, whereas microglia play a crucial role in selectively removing excess myelin through phagocytosis to regulate myelin refinement. It is dispensable for developmental myelin ensheathment, although during development, phagocytosis by microglia does not trigger an inflammatory response [128]. However, they are required for the subsequent regulation of myelin growth and associated cognitive function and for the preservation of myelin integrity by preventing myelin degeneration [129]. In this process, extra-cellular ATP may guide microglia toward or away from target neurons, which aids in the migration of microglia [130]. Studies on Alzheimer’s disease have suggested that neuroinflammation can impede the ability of OLGs to produce myelin, affecting myelin regeneration [131]. When the myelin sheath is damaged, it needs to be removed for regeneration to occur.

Mounting evidence suggests that microglia, which are professional phagocytes of the CNS, are responsible for clearing myelin debris through the autophagy‒lysosome pathway during demyelination [132]. This function significantly impacts the generation of OLGs. Defective spleen tyrosine kinase (SYK) signaling in microglia during demyelinating disease causes damaged myelin debris accumulation and impaired OLGs proliferation [133], and the aryl hydrocarbon receptor (AhR) in microglia regulates the expression of SYK, enhancing the phagocytic function of microglia and promoting myelin regeneration [ 134– 136]. Fasudil enhances the phagocytosis of myelin debris by microglia by activating the TREM2/DAP12 pathway [137]. Microglia are responsible for clearing myelin debris and promoting myelin regeneration, whereas astrocytes play a role in recruiting microglia to the site of demyelination [6]. CX3CR1 mediates the phagocytosis of single dying oligodendrocytes by microglia, which can perform this process with remarkable precision and speed [138].

Steroids play crucial roles in promoting the formation and repair of myelin [139]. In EAE models of MS, metabolic pathways involving cholesterol in microglia, particularly cholesterol synthesis, have been identified as critical components of remyelination. The persistent pro-inflammatory activation of microglia with squalene synthase dysfunction causes the arrest of OLGs maturation and poor remyelination [140]. Recent studies indicate that OLGs and OPCs not only are passive targets but also actively participate in MS; however, the exact mechanism of the interaction between OLGs and microglia and how they co-regulate the immune response during demyelination disease are still not fully understood [141].

### Interaction between OLGs and neurons

OLGs play crucial roles in supporting neuronal function. They interact with neurons to maintain the healthy state of the CNS, making them lifelong companions of neurons. Furthermore, the release of growth factors by neurons influences the differentiation of OLGs, which also contributes to the process of myelination [6].

#### OLGs modulate neuronal function

OLGs provide basic metabolic support for neurons. By expressing specialized transporters, these cells deliver glucose-derived metabolites to neurons through cytoplasmic myelin channels and monocarboxylic acid transporters. This process enables short carbon chain energy metabolites, such as pyruvate, to be transported to neurons, providing them with the necessary energy for proper functioning [11]. OLGs release signaling factors to neurons in the process of myelination to regulate neuronal signal transduction [21]. Fibroblast growth factor 2 (FGF2) is secreted by OPCs and helps to maintain excitatory glutamatergic neurotransmission [142]. Jang *et al* . [143] reported that OLGs modulate neurotransmitter release at presynaptic terminals through the secretion of BDNF. BDNF in the OLGs activates TrkB receptors to ensure fast and reliable neurotransmitter release and auditory transmission in the developing brain. Myelin produced by OLGs consists of several proteins that influence neuroregeneration. Nogo-A can interact with neurons via two main termini, the amino-Nogo terminus and the Nogo-66 terminus, and remyelination is observed when this inhibitor is antagonized [144]. Overall, OLGs are vital for both the structural and functional support of neurons in the CNS, underlining the critical glioneuronal interactions that facilitate neural communication and metabolic exchange.

The exosomes released by OLGs respond to neuronal signals and exhibit neuroprotective properties [73]. GABA is an important neurotransmitter released by neurons that increases the neuronal membrane potential, AP production, and zinc transmembrane transport. OLGs are involved in regulating GABAergic neurons excitability and synaptic function [20]. Studies have shown that the selective activation of NG2 glial cells via an optogenetic model induces GABA neurotransmitter release, thereby specifically modulating the postsynaptic inhibitory activity of adjacent interneurons in the adult mouse hippocampus [145]. Exosomes also play important roles in axon–glial cell communication. Neurotransmitter glutamate regulates the exosome secretion of OLGs by activating glial ionogenic glutamate receptors. In turn, the neurons internalize the exosomes released from the OLGs and retrieve their cargo. Under stress condition, the supply of cultured neurons with OLGs exosomes supports neuronal metabolism and increases neuronal viability [146]. Neurons are particularly vulnerable to oxidative stress [147]. Disruption of iron regulation in the brain is involved in the pathogenesis of various neurodegenerative diseases [148]. OLGs play a crucial role in protecting neurons from iron-induced toxicity by secreting ferritin heavy chains as part of their antioxidant defense system. When the release of extracellular vesicles in OLGs or the expression of ferritin heavy chains is disrupted, mice experience neuronal loss and oxidative damage [149]. In summary, OLGs and their exosomes communicate with neurons in various ways, influencing neurotransmission, myelination, and neuronal survival. These findings highlight their therapeutic potential in neurodegenerative disorders.

#### The modulatory effects of neuronal activity on myelin

The Tau protein plays a crucial role in the myelination process, as it directly supports the extension, wrapping, and myelination of OLGs. Significant amounts of Tau protein are present in the axon during its establishment. It has been confirmed that any changes in myelin can affect the function of neurons. Additionally, an imbalance of Tau subtypes can reduce the function of myelin, which means that it cannot adapt to the function of neurons. This can lead to the death of the OLGs-neuronal unit, ultimately affecting the overall health of neurons [150].

Although neuronal activity does not play a direct role in myelination, it does not affect the adaptive response following myelination, which affects the size and amount of myelin [ 151, 152]. In certain axon subtypes and regions of the nervous system, neuronal activity not only regulates the production of OLGs but also induces structural changes in the myelin sheath. Neuronal activity triggers the rapid proliferation of neural progenitor cells and OPCs in specific areas, promoting the formation of OLGs, which accelerates myelin formation and increases the thickness of the myelin sheath [153]. However, abnormal increases in neural circuit activity can lead to maladaptive myelination, potentially contributing to the development of various glioma types through altered paracrine signaling and neuron-to-glioma synaptic interactions [154]. Thus, neuronal activity exerts a nuanced influence on myelination, with implications for both normal CNS function and the pathogenesis of gliomas. Further research is needed to better understand the mechanisms involved and explore potential therapeutic interventions.

#### Selective myelination by OLGs

OLGs do not play the same role in all neurons, and this role is somewhat selective, but they have a clear bias toward the type of axon that forms myelin. Different strategies are applied to different types of neurons during myelination. OLGs do not passively accept any axons in their vicinity but instead target axons of specific types, likely through active signals or molecules intrinsic to specific neuronal subtypes. Some OLGs disproportionately myelinate the axons of inhibitory interneurons, whereas others primarily target excitatory axons, and some show no bias [21]. OLGs and OPCs display heterogeneity in the brain [ 155– 157], with different subpopulations of OLGs showing varying preferences in myelination. Some OLGs myelinate axons of both excitatory and inhibitory neurons equally, whereas others exhibit a bias toward one type. Pyramidal neurons of different cortical layers differentially influence the distribution of myelinating OLGs myelin formation [158]. The specific interaction of OLGs with specific neuronal subtypes during myelination not only reflects the complexity of CNS wiring but also presents opportunities for precision medicine approaches in neurological disorders.

## Conclusions and Perspectives

In the CNS, not all neurons are myelinated equally. The pattern of myelination varies depending on the localized area of the neuron [158]. Epigenetic factors are thought to primarily influence these variations in myelination patterns. The program that guides myelination is established before differentiation, with the potential for adaptive myelination processes to occur throughout or after differentiation [151]. Synaptic activity and myelination are not synchronized. Synapses are involved in regulating the early development of NG2 glial cells, preparing them for subsequent myelination [159]. Scholars have identified a subgroup of OLGs termed disease-associated OLGs (DOL), which are implicated in brain pathology. In mouse models of amyloidosis, DOL response to CNS damage occurs long after the accumulation of amyloid plaques [160]. Studies have shown that signals from the systemic environment can counteract age-related intrinsic changes within OPCs. For example, transplanted young macrophages are more effective than aged macrophages in improving wound repair in elderly animals. Exposure to a young systemic environment can enhance myelin regeneration in elderly animals [161]. The age-related decline in OPCs differentiation may be attributed to changes in axonal metabolic characteristics of axons, which play a critical role in this process [32]. All evidence suggests that the activity of OLGs and the myelination process are time sensitive.

The formation of myelin in the neocortex is a complex process that involves coordinated interactions between OLGs and neurons, along with other glial cells. However, this interaction is not static and varies in time and space. On one hand, glial cells and neurons are heterogeneous in the brain [162], and the exchange of information between different cellular subgroups is selective. On the other hand, in the course of disease, new cellular subgroups emerge, and the addition of new members inevitably alters the original cellular interaction patterns. Studies have shown that administering drugs transiently, rather than continuously, can be more effective in treating demyelination diseases over a longer period of time. The timing of treatment is crucial, as incorrect timing can lead to more severe demyelinating lesions [132]. This implies that the traditional method of selecting a single time point to study myelin mechanisms is flawed. As the field of cell research has advanced, it has become increasingly clear that a simple understanding of cellular processes is not sufficient to fully comprehend physiological and pathological mechanisms. Marques *et al* . [163] performed single-cell RNA-Seq on 5072 cells of the oligodendrocyte lineage from ten regions of the mouse juvenile/adult CNS and reported that OLGs already exhibit rich diversity in the cellular population at the stage of myelin formation. Hou *et al*. [162] demonstrated via single-nucleus RNA sequencing that a population of demyelination-associated oligodendrocytes is dependent on a TREM2-mediated microglial response during demyelination and remyelination induced by cuprizone. Using transcriptomic data, Enrich-Bengoa *et al*. [164] elucidated the genetic crosstalk between microglia and OPCs during demyelination and remyelination, and their cellular crosstalk analysis identified novel markers for microglial ligands, OPCs receptors, and target genes. These studies have confirmed the potential of various sequencing technologies in investigating cell interactions in the context of myelination and disease environments. In the future, advancements in spatial transcriptomics, combining super-resolution imaging and sequencing technologies, along with the establishment of enduring models, will provide new opportunities for a more comprehensive understanding of biological systems [165]. In addition, Fagiani *et al*. [166] proposed an organoid platform in which organoids properly mimic the macroglia–microglia neurodegenerative phenotypes and intercellular communication observed in chronic active MS. Barreras *et al*. [167] developed an iPSC-derived 3D human brain model, and the model presented evidence of neuron–neuron and neuron–glia interactions. Hyung *et al* . [168] presented a three-dimensional peripheral nervous system microfluidic platform that recapitulates the full spectrum of myelination, demyelination, and remyelination via primary Schwann cells and motor neurons. These findings support the implementation of this organoid model for drug screening to halt inflammatory neurodegeneration.

Demyelinating diseases are characterized by the loss of myelin, which leads to a variety of symptoms. Owing to the complex and diverse nature of the CNS, targeting a single therapeutic site or mechanism has limitations in both experimental and applied applications. OLGs are key players in all stages of demyelinating diseases, as they are responsible for producing myelin. Therefore, therapeutic research targeting OLGs is the most effective and direct approach. To improve the therapeutic effects of treatments for demyelinating diseases, it is necessary to conduct further research on the mechanisms of OLGs and explore various treatment methods involving multiple mechanisms and sites. This review focuses on the interactions between OLGs and other cells, signal transduction pathways, and gene and protein expressions. Understanding the interaction between OLGs and other glial cells will help reduce neuroinflammation caused by demyelinating diseases, prevent neuronal death, and create a suitable microenvironment for remyelination. By utilizing multiple therapeutic approaches, it is possible to treat demyelinating diseases more effectively and promote repair and functional recovery of the nervous system.

Glial cell crosstalk is a crucial factor in CNS diseases. In this paper, we reviewed the interactions between OLGs, other glial cells, and neurons in demyelinating diseases. By exploring the mechanism and signaling pathway of crosstalk between OLGs and other cells, we highlight the importance of their interactions. In addition, we suggest that the interaction between glial cells has certain spatial and temporal characteristics and that the mapping of space and time is crucial for studying cell interactions. These findings provide a more macro understanding of the CNS and are highly important for the treatment of demyelinating diseases.

## Supporting information

Supplementary_File
