# Supplementary material for: Oligodendrocytes interactions with glial cells and neurons in demyelinating disease: Oligodendrocyte interactions in demyelination
Source: Acta Biochim Biophys Sin (Shanghai). 2025 Aug 13;57(12):1909–22. doi: 10.3724/abbs.2025105 (PMC12747983; doi:10.3724/abbs.2025105)
Supplement: Supplementary_File [file Supplementary_File.pdf]

THE LICENSE AGREEMENT

License of publishing rights
I hereby grant to **Center for Excellence in Molecular Cell Science, CAS** an exclusive publishing and distribution license in the manuscript identified above and any tables, illustrations or other material submitted for publication as part of the manuscript (the "Article") in print, electronic and all other media (whether now known or later developed), in any form, in all languages, throughout the world, for the full term of copyright, and the right to license others to do the same, effective when the Article is accepted for publication. This license includes the right to enforce the rights granted hereunder against third parties.

Supplemental Materials
"Supplemental Materials" shall mean materials published as a supplemental part of the Article, including but not limited to graphical, illustrative, video and audio material.

With respect to any Supplemental Materials that I submit, **Center for Excellence in Molecular Cell Science, CAS** shall have a perpetual worldwide, non-exclusive right and license to publish, extract, reformat, adapt, build upon, index, redistribute, link to and otherwise use all or any part of the Supplemental Materials, in all forms and media (whether now known or later developed) and permit others to do so.

Scholarly communication rights
I understand that I retain the copyright in the Article and that no rights in patents, trademarks or other intellectual property rights are transferred to the **Center for Excellence in Molecular Cell Science, CAS**. As the author of the Article, I understand that I shall have the same rights to reuse the Article as those allowed to third party users (and **Center for Excellence in Molecular Cell Science, CAS**) of the Article under the CC BY License, CC BY-NC License, or CC BY-NC-ND License.

Reversion of rights
Articles may sometimes be accepted for publication but later rejected in the publication process, even in some cases after public posting in "Articles in Press" form, in which case all rights will revert to the author.

Revisions and addenda
I understand that no revisions, additional terms or addenda to this License Agreement can be accepted without **Center for Excellence in Molecular Cell Science, CAS**'s express written consent. I understand that this License Agreement supersedes any previous agreements I have entered into with *Journal Owner* in relation to the Article from the date hereof.

Copyright Notice
The publisher shall publish and distribute the Article with the appropriate copyright notice.

Author Representations/Ethics and Disclosure
I affirm the Author Representations noted below, and confirm that I have reviewed and complied with the relevant Instructions to Authors, Ethics in Publishing policy, and Declarations of Interest disclosure. Please note that some journals may require that all co-authors sign and submit Declarations of Interest disclosure forms. I am also aware of the publisher's policies with respect to retractions and withdrawal.

Author Representations
- The Article I have submitted to the journal for review is original, has been written by the stated authors and has not been previously published.
- The Article was not submitted for review to another journal while under review by this journal and will not be submitted to any other journal.
- The Article and the Supplemental Materials do not infringe any copyright, violate any other intellectual property, privacy or other rights of any person or entity, or contain any libelous or other unlawful matter.
- I have obtained written permission from copyright owners for any excerpts from copyrighted works that are included and have credited the sources in the Article or the Supplemental Materials.
- Except as expressly set out in this License Agreement, the Article is not subject to any prior rights or licenses
- If I am using any personal details or images of patients, research subjects or other individuals, I have obtained all consents required by applicable law and complied with the publisher's policies relating to the use of such images or personal information.
- Any software contained in the Supplemental Materials is free from viruses, contaminants or worms.
- If the Article or any of the Supplemental Materials were prepared jointly with other authors, I have informed the co-author(s) of the terms of this License Agreement and that I am signing on their behalf as their agent, and I am authorized to do so.

LICENSE AGREEMENT

Dear author,

We need your written permission before we can publish your accepted article in *Acta Biochimica et Biophysica Sinica* on behalf of Center for Excellence in Molecular Cell Science, CAS. Please read this form carefully. If you are happy with its terms complete it and immediately email a copy to abbs@sibs.ac.cn.

As the open access article, you will be charged an article-publishing charge (APC). After you sign the license, the editorial office will contact you.

PLEASE PROVIDE US WITH THE FOLLOWING INFORMATION, REVIEW OUR POLICIES AND THE LICENSE AGREEMENT, AND INDICATE YOUR ACCEPTANCE OF THE TERMS

To be published in the journal: Acta Biochimica et Biophysica Sinica Manuscript number: ABBS-2024-981.R2

Article entitled: Oligodendrocytes Interactions with Glial Cells and Neurons in Demyelination

Corresponding author: Xing Li, Yuan Zhang

Please select which open access license you would like to use, review the License Agreement, and then sign and date the License Agreement in black ink.

☑ CC BY: Once published, your Article will be distributed under the terms of the Creative Commons Attribution License (https://creativecommons.org/licenses/by/4.0/) which permits unrestricted use, distribution, and reproduction in any medium, provided the original work is properly cited.

☑ CC BY-NC: Once published your Article will be distributed under the terms of the Creative Commons Attribution Non-Commercial License (https://creativecommons.org/licenses/by-nc/4.0/) which permits non-commercial use, distribution, and reproduction in any medium, provided the original work is properly cited.

☑ CC BY-NC-ND: Once published your Article will be distributed under the terms of the Creative Commons Attribution-NonCommercial-NoDerivs License (https://creativecommons.org/licenses/by-nc-nd/4.0/) which permits non-commercial use, distribution, and reproduction in any medium, provided the original work is not altered or transformed in any way and the original work is properly cited.

Signed: Xing Li Name printed: Xing Li

Title and Company (if employer representative): Professor of College of Life Sciences

Date: April 23, 2025
